# Comparative transcriptomics in alternate bearing cultivar Dashehari reveals the genetic model of flowering in mango

**DOI:** 10.3389/fgene.2022.1061168

**Published:** 2023-01-10

**Authors:** Harmanpreet Kaur, Gurupkar Singh Sidhu, Amandeep Mittal, Inderjit Singh Yadav, Meenakshi Mittal, Deepak Singla, Navprem Singh, Parveen Chhuneja

**Affiliations:** ^1^ School of Agricultural Biotechnology, Punjab Agricultural University, Ludhiana, India; ^2^ Department of Plant Breeding and Genetics, Punjab Agricultural University, Ludhiana, India; ^3^ Department of Fruit Science, Punjab Agricultural University, Ludhiana, India

**Keywords:** Dashehari mango, alternate bearing, floral development, RNA-seq, flowering genes

## Abstract

Flowering is a complex developmental process, with physiological and morphological phases influenced by a variety of external and internal factors. Interestingly, many mango cultivars tend to bear fruit biennially because of irregular flowering, and this has a negative impact on mango flowering and the subsequent yield, resulting in significant economic losses. In this article, transcriptome analysis was carried out on four tissues of mango cv. Dashehari (bearing tree leaf, shoot apex, inflorescence, and non-bearing tree leaf). *De novo* transcriptome assembly of RNA-seq reads of Dashehari using the Trinity pipeline generated 67,915 transcripts, with 25,776 genes identified. 85 flowering genes, represented by 179 transcripts, were differentially expressed in bearing *vs.* non-bearing leaf tissues. Gene set enrichment analysis of flowering genes identified significant upregulation of flowering related genes in inflorescence tissues compared to bearing leaf tissues. The flowering genes *FT*, *CO*, *GI*, *ELF 4*, *FLD*, *FCA*, *AP*1, *LHY*, and *SCO1* were upregulated in the bearing leaf tissues. Pathway analysis of DEGs showed significant upregulation of phenylpropanoid and sucrose and starch pathways in non-bearing leaf tissue compared with bearing leaf tissue. The comparative transcriptome analysis performed in this study significantly increases the understanding of the molecular mechanisms driving the flowering process as well as alternative bearing in mango.

## 1 Introduction

Mango (*Mangifera indica* L.) is one of the most important tropical fruits worldwide and is known as the king of fruits (Singh, 2016). It is a dicotyledonous fruit belonging to the family Anacardiaceae and the order Sapindales. Its centre of origin is the Indo-Burmese region. It is widely distributed in South Asia and has become one of the most cultivated fruit crops, with substantial economic importance and high commercial value. Flowering is one of the major physiological events in mango, preceded by the differentiation of flower buds. This process begins with shoot initiation followed by the differentiation of flower buds and the fruit set. The density of flowering varies according to different factors such as cultivar, age of a tree, condition of the environment, and growth conditions in humid or dry tropics. Flowering also depends on the age of the last vegetative flush (Ramírez and Davenport, 2010).

Mango fruit crops have a strong tendency toward alternate or biennial bearing. Mango is a terminal bearer, with the phenomenon underlying the switch from vegetative to reproductive mode poorly understood. In India, poor yield is commonly associated with the alternation in bearing. Different reasons for this bienniality include the exhaustion of trees during heavy crop load and vigorous vegetative crop growth. A heavy fruit load in one year induces a decrease in the formation of flower buds and lower or no production in the succeeding year (Rani, 2018), giving rise to the biennial rhythm of “on” and “off” years, or, in other words, heavy and poor bearing years. This is a serious problem for growers, leading to major economic losses.

A less understood substance known as “florigen” or “florigenic promoter” (FP) is synthesized in leaves and is responsible for flowering. FP movement can reach up to 100 cm from one branch to another (Ramírez and Davenport, 2010). The flowering phenomenon in mango is very complex and is challenging for physiologists, breeders, and growers since flowering at a favorable time provides a desirable crop yield (Sandip et al., 2015). Moreover, in unfavorable seasons, flowering may lead to yield loss owing to inadequate growth of photosynthetic organs or poor fertility due to heat/cold stress during reproduction. Hence, understanding the flowering mechanism can contribute to the development of novel practices in mango breeding to obtain regular bearing crops and optimum yields (Tsuji, 2017). Therefore, it is important to understand floral induction and bearing habit to ensure regular flower bud differentiation, as these are prerequisites for annual steady production.

A number of conditional transcriptomes in mango have been developed in the last decade (Bajpai et al., 2018; Luria et al., 2014; Deshpande et al., 2017; Tafolla-Arellano et al., 2017; Yadav et al., 2020; Liang et al., 2022). Luria et al. (2014) developed a transcriptome assembly for mango fruit peel. The authors discovered three categories of genes that were highly expressed during hot water bath (HWB) treatment: Genes related to biotic and abiotic stress responses, genes linked with chlorophyll breakdown and photosynthesis, and genes involved in sugar and flavonoid metabolism, suggesting a molecular basis for the biochemical and physiological effects of postharvest HWB treatment. Tafolla-Arellano et al. (2017) studied the transcriptome of mango in epidermal fruit peel, identifying putative genes associated with a cuticle. This suggested a pathway for the biosynthesis of the cuticle component, cutin, which over-accumulated during overripening. In mango *cv.* Alphonso, Deshpande et al. (2017) studied transcriptional transitions at seven stages of fruit growth and ripening, leading to an understanding of flavonoids, β-carotene, α-tocopherols, and terpenoid backbone biosynthesis. Bajpai et al. (2018) identified candidate genes related to anthocyanin development in mango. Liang et al. (2022) identified different pathways and metabolites using comparative transcriptomics and metabolomics of flowering and never-flowering Chinese mango cultivars. However, no studies to date have examined the phenomenon of alternate bearing in mango. Thus, this study was conducted to understand alternate bearing in mango.

Cultivars such as Dashehari, Langra, Chausa, and Amrapali are preferred for cultivation in north India. Dashehari, whose name is derived from the village Dashehari near Lucknow, is one of the most grown cultivars. Dashehari has a small to medium fruit size, oblong oblique shape, and a yellow fruit color. The fruit quality is excellent and the storage quality is good. It is a mid-season variety and is mainly used for table purposes. Compared with other monocots such as rice, wheat, and cereal, much less information is available regarding the flowering genes that regulate the vegetative to flowering transition and flower initiation in mango. In the present study, we report *de novo* transcriptome assembly and functional annotation of mango cv. Dashehari and comparative RNA-seq analysis of inflorescence, shoot apex, leaf of bearing plant, and leaf of non-bearing plant tissues. The major objective of the study is the identification of flowering switch factors in “on” *vs.* “off” years in the mango plant.

## 2 Materials and methods

### 2.1 Plant material

**FIGURE 1:**
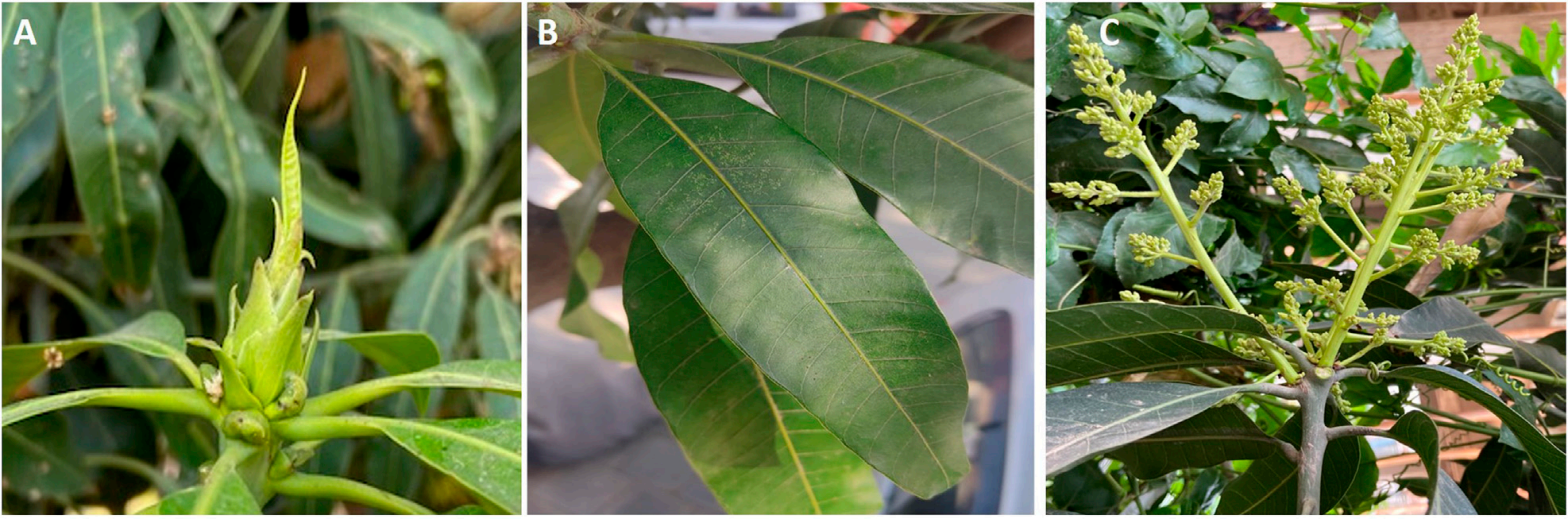
Dashehari mango samples used for RNA isolation: **(A)** shoot apex; **(B)** immediate mature leaf (within 50 cm of the shoot apex); and **(C)** inflorescence.

Fully mature dark green leaf, inflorescence, and shoot apex (shoot tip from current year flowering branch) were collected from 10-year-old bearing and non-bearing Dashehari mango trees (Figure 1) that were grown in 2018 using uniform cultural practices. Dashehari were clonally propagated and raised in the mother block of the Regional Fruit Research Station-Gangian, Hoshiarpur, Punjab, India. The samples were flash frozen in liquid nitrogen and stored at −80°C until processing for total RNA extraction.

### 2.2 RNA extraction and sequencing

Total RNA from the leaves, inflorescence, and apex of bearing Dashehari tree was extracted using a Spectrum™ Plant Total RNA Kit (Sigma-Aldrich) as per Mittal et al. (2020). RNA quantity and quality were analyzed using 1.2% agarose gel electrophoresis. The RNA samples were further analyzed using a Bioanlyser and samples with RIN > 7 were used for preparing the 100 bp PE libraries, with an average insert size of 300 bp. Libraries pertaining to tissues, viz., bearing Dashehari leaf, inflorescence, shoot apex, and non-bearing leaf were sequenced on an Illumina HiSeq2500 platform.

### 2.3 Data pre-processing, *de novo* assembly, and quality estimation

The quality of reads was assessed using the FASTQC toolkit (Patel and Jain, 2012) and reads were cleaned with Trimmomatic (Bolger et al., 2014). The details of the six libraries, corresponding to four tissue types, are provided in Supplementary Table S1. *De novo* assembly of Dashehari RNA-seq data was performed using Trinity (Haas et al., 2013). The quality of assembly was checked with Benchmarking Universal Single-Copy Orthologs (BUSCO) (Waterhouse et al., 2018), using eudicot genes as the reference set.

### 2.4 Differential gene expression and functional annotation

The uniquely mapped reads of the reference transcriptome were used to calculate the normalized expression levels (FPKM: fragments per kilobase of transcript, effective length per million fragments mapped to all transcripts) of each sample. RSEM software was used to calculate the count values of the transcripts for each sample, with and without biological replicates (Li and Dewey, 2011). To normalize data from different libraries, Trimmed Mean of M-values (TMM) normalization was performed in Trinity. The expected count values thus obtained were analyzed using edgeR (Robinson et al., 2009) to identify the differentially expressed gene transcripts/isoforms (DEG), at a false discovery rate (FDR) of <0.1. Transcripts showing at least a 4-fold log2 change in values and a false discovery-corrected statistical significance of *p* < 0.0001 were considered to be differentially expressed.

The functional annotation of the identified gene/isoforms, based on homology, was identified against the “nr” database in blastx. Protein families were assigned by searching the Protein family (Pfam) database using the HMM-based tool PfamScan. Blast2GO (Conesa and Götz, 2008) was used for gene ontology assessment and transcripts were categorized based on biological processes, cellular component, and molecular function. Using the KEGG Automatic Annotation Service (KAAS) (Kanehisa and Goto, 2000) and Plant Reactome, the assembly was mapped to classical reference pathways in KEGG in order to identify the biological processes.

### 2.5 cDNA synthesis and quantitative real time-PCR (qRT-PCR) assay

Three micrograms of total RNA extracted from the leaf, apex, and inflorescence of mango-bearing trees and the leaf of non-bearing Dashehari trees was treated with DNAase I using a Spectrum™ Plant Total RNA Kit (Sigma-Aldrich Kit). RNA integrity was analysed using 1.2% agarose denaturing formaldehyde gel (Supplementary Figure S1), followed by cDNA synthesis with a Maxima cDNA synthesis kit (Thermo Scientific^®^). The expression of genes linked to the flowering mechanism was examined using the Applied Biosystems™ Quant Studio 5 Real-time PCR, with measurements performed in triplicates, employing SYBR green as a reporter (Supplementary Table S2). For amplification, the following reaction conditions were used: initial incubation at 50°C for 2 min, 95°C for 2 min, 40 cycles of denaturation at 95°C for 10 s and at 60°C for 30 s, followed by melt curve analysis at 95°C for 15 s, 60°C for 1 min, and 95°C for 15 s. Gene expression was calculated using the 2^−ΔΔCT^ approach (Livak and Schmittgen, 2001).

## 3 Results

### 3.1 Transcriptome assembly

**FIGURE 2:**
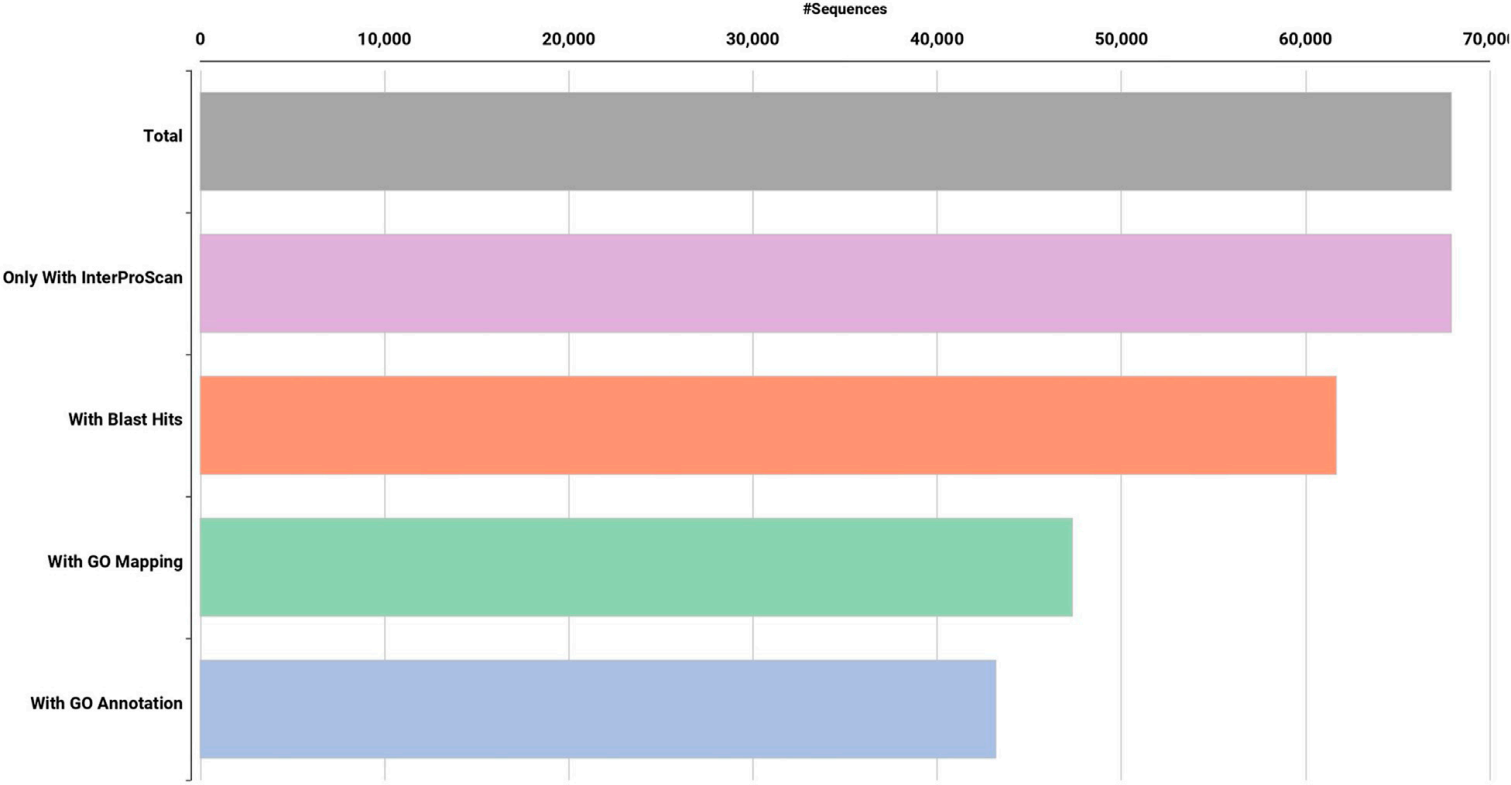
Functional annotation of *de novo* Dashehari assembly using the non-redundant (nr) database.

High quality (74%–78%) Dashehari *de novo* transcriptome assembly was developed using more than 134 M surviving paired end RNA-seq reads of Q > 30 from libraries of the bearing leaf (34.5 M), inflorescence (25.0 M and 28.9 M), apex (28.8 M), non-bearing leaf (29.8 M and 27.1 M), and malformed inflorescence (Kaur et al., unpublished) using the Trinity pipeline. The N_50_ value evaluates the contiguity of the assembled sequences and is defined as the maximum length whereby ≥50% of the total assembled sequence resides in contigs of at least that long. An N_50_ value of 1,981 was obtained for the present transcriptome *de novo* assembly, and a total of 25,776 Trinity genes and 67,915 transcripts were obtained (Supplementary Data Sheet; Datafile_1_Assembly annotation and Table 1). Out of 67,915 transcripts, 43,216 exhibited the tag for blasted, mapped, and annotated, 4156 transcripts for blasted and mapped, and 6,207 transcripts displayed only blast within the database. However, 14,336 showed no blast result (Figure 2).

**TABLE 1:** Assembly statistics of the Dashehari transcriptome.

Dashehari transcriptome *de novo* assembly	Statistics
Total transcripts	67,915
Total genes	25,776
Per cent GC	42.2
N_50_ value	1,981
Total assembled bases (all transcripts)	96,486,409
Total assembled bases (longest isoform per gene)	28,520,127
Benchmarking Universal Single-Copy Orthologs (BUSCOs)	
Complete BUSCOs (C)	1,901 (89.6%)
Complete and single-copy BUSCOs (S)	1,029 (48.5%)
Complete and duplicated BUSCOs (D)	872 (41.1%)
Fragmented BUSCOs (F)	65 (3.1%)
Missing BUSCOs (M)	155 (7.3%)
Total BUSCO groups searched	2,121

**FIGURE 3:**
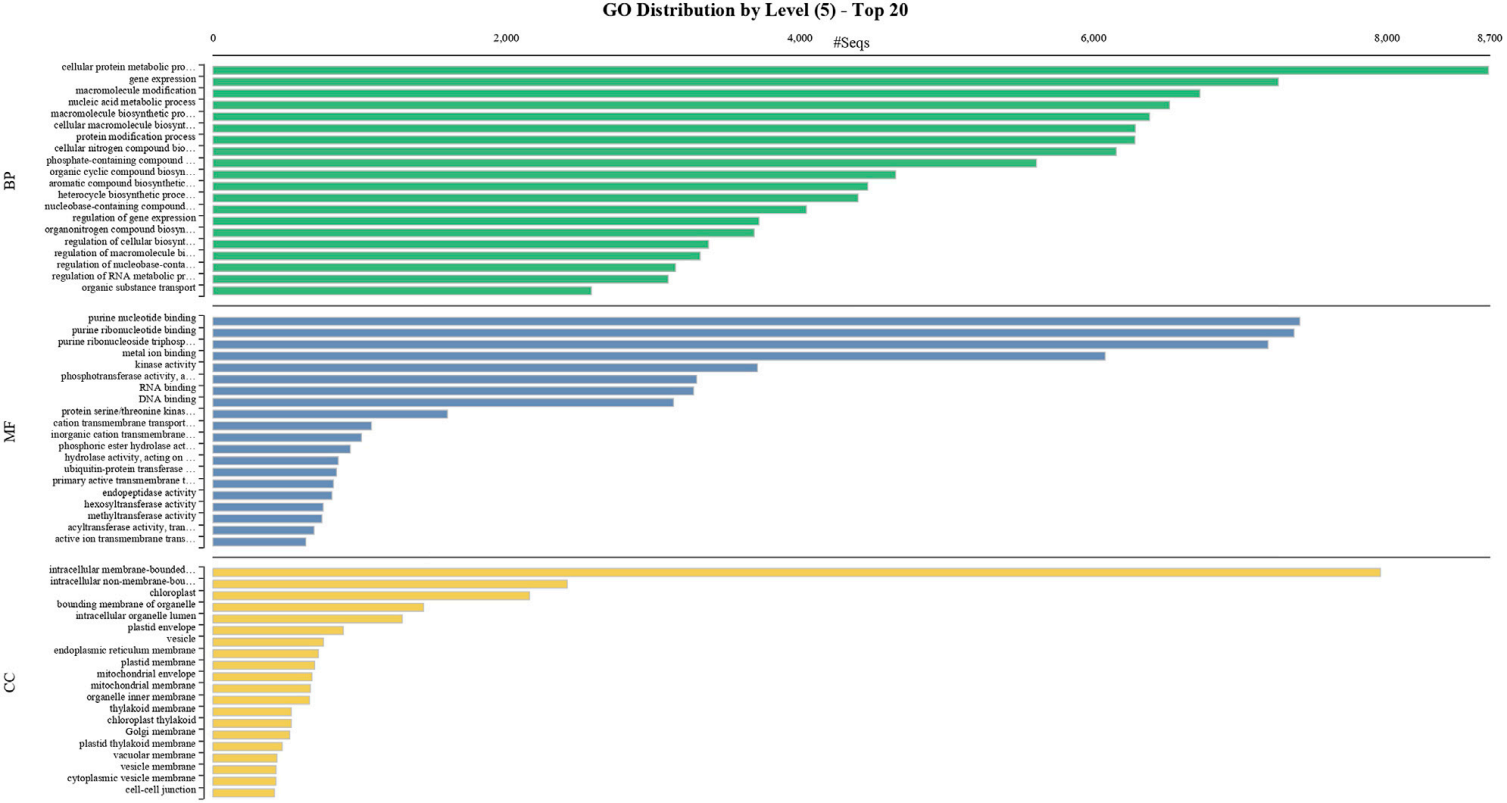
GO classification of the annotated Mango transcripts categorized into cellular component, molecular function, and biological process.

**FIGURE 4:**
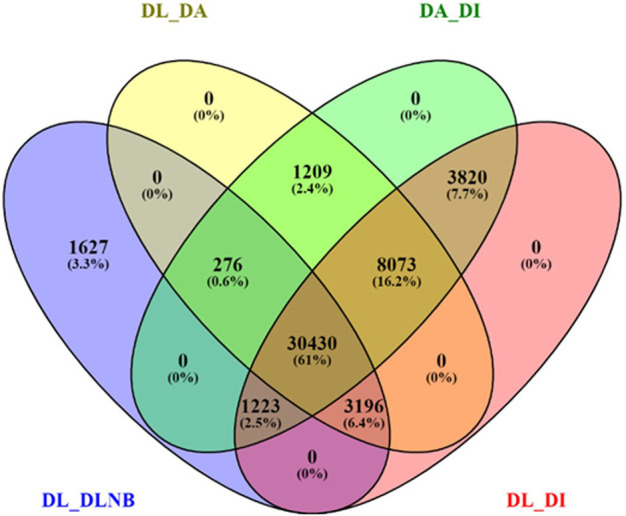
Venn diagram of differentially expressed genes with different comparisons of the bearing and non-bearing leaf, apex, and inflorescence. DL—Dashehari bearing leaf; DLNB—Dashehari non-bearing leaf; DA—Dashehari shoot apex; DI—Dashehari Inflorescence.

BUSCO analysis with eudicots identified 89.6% complete BUSCO genes, implying the good quality of the assembly (Table 1). A gene ontology assessment with Blast2GO allowed assignment of gene ontology terms to 46,866 transcripts, where the biological process consists of 33,783 transcripts, cellular components of 4792, and molecular function of 8291 (Figure 3). A protein family search identified 36,657 protein domains, 27,342 families, and 595 motifs. The genes were annotated with Diamond BLAST, against the “nr” database (Supplementary Data Sheet; Datafile_1_Assembly annotation) and protein families were scanned with PfamScan. To identify common transcripts related to flowering, a Venn diagram was generated from upregulated and downregulated genes in DL_DLNB (Figure 4). Comparisons of the leaf *vs.* inflorescence (DL_DI), inflorescence *vs.* apex (DI_DA), and leaf *vs.* apex (DL_DA) were performed to understand the development in Dashehari. To understand the bearing habit in Dashehari, the bearing leaf was compared with the non-bearing leaf (DL_DLNB). In DL_DLNB, a total of 37,236 transcripts were identified as differentially expressed, of which 1,603 were upregulated and 955 were downregulated ≥4 fold Supplementary Table S3; Supplementary Data Sheet; Datafile_2_DL_DLNB, and Supplementary Figure S2). Additionally, 38,579 differentially expressed transcripts were identified in leaf *vs.* apex tissues (DL_DA), with 7,923 significantly upregulated in the leaf and 8,790 significantly downregulated (Supplementary Data Sheet; Datafile_3_DL_DA). Comparing the inflorescence with the leaf and apex, 7,773 and 1,794 differentially expressed genes were upregulated while 6,143 and 263 genes were downregulated, respectively (Supplementary Table S3; Supplementary Data Sheet; Datafile_4_DL_DI and Datafile_5_DA_DI). A heat map based on expression patterns of flowering related genes is shown in Supplementary Figure S3.

### 3.2 Flowering genes differentially expressed in apex and inflorescence, compared with bearing leaf

**FIGURE 5:**
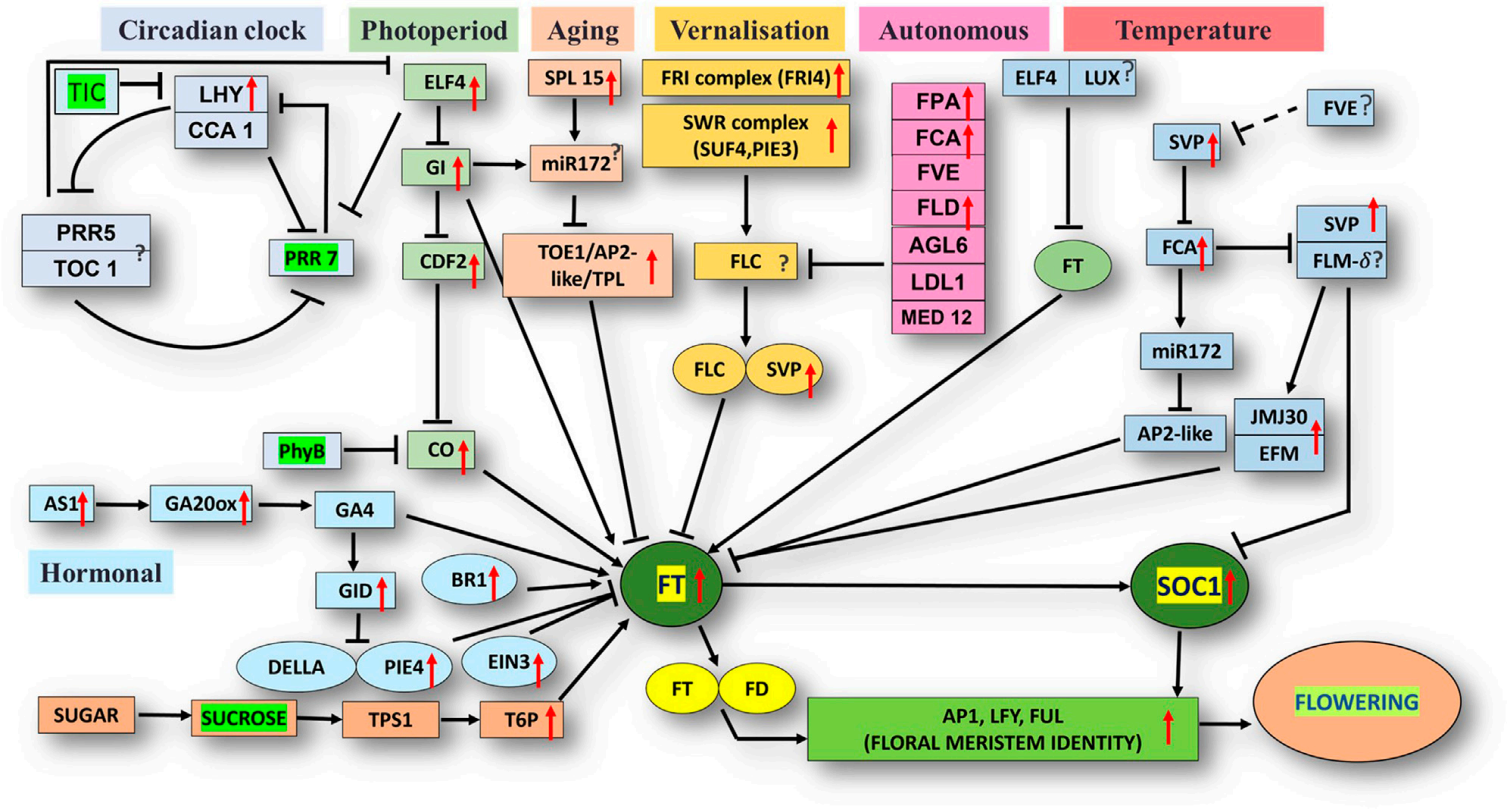
Flor-ID flowering genes—binned into circadian clock, photoperiod, aging, vernalization, autonomous and temperature related pathway—expressed in bearing *vs.* non-bearing Dashehari leaf tissue.

The unigene sequence of the mango transcriptome was blasted with the *Arabidopsis thaliana* database, i.e., FLOR-ID, and based on this, annotations of flowering related genes involved in different pathways were identified (Figure 5).

Major genes such as *FRIGIDA*, *VIN3*, *APETALA1-like* protein, *UPSTREAM OF FLC, FTIP1*, *EMBRYONIC FLOWER*, *UNUSUAL FLORAL ORGANS-like*, *SPL15* genes, and *FLC EXPRESSOR-like* exhibited higher expressions in the apex and inflorescence. However, expressions of the genes related to *GIGANTEA* (*GI*), MADS-box protein *SOC1-like*, *SVP-like*, and *LHY* were upregulated in the leaf. Transcripts of phytochrome B (PHYB), which is involved in the photoperiod pathway, were also highly expressed in the leaves as compared to the inflorescence and apex. On the contrary, when apex and inflorescence were compared 1,794 transcripts were upregulated and 263 downregulated in the inflorescence (Supplementary Data Sheet; Datafile_5_DA_DI). *CONSTANS* (*CO*), *UNUSUAL FLORAL ORGANS-like*, *FTIP-1*, *APETALA1-like*, *EMBRYONIC FLOWER*, and others exhibited higher expressions in the apex. However, in the inflorescence, *SUF4*, *gibberellin receptor GID1B-like*, *TPL*, *FLD*, and *SEPALLATA3*-like expressions were higher. Interestingly, the expression of *FLOWERING LOCUS T* (*FT*) was higher in the apex and inflorescence than in the leaf, in line with the understanding that the florigen transcript was generated in the leaves and subsequently transported to the apex and inflorescence to support floral transition (Corbesier et al., 2003; Putterill and Varkonyi-Gasic, 2016).

### 3.3 Floral identity, vernalization, photoperiod, aging, circadian clock, and hormone signalling pathway genes differentially expressed in bearing *vs.* non-bearing mango leaf

Differential expression analysis of the bearing *vs.* non-bearing mango leaf identified 1,603 transcripts upregulated and 955 downregulated in the non-bearing leaf (Supplementary Table S3). Floral meristem identity genes such as *APETALA1* (*AP1)*, MADS-box transcription factors *SUPPRESSOR OF OVEREXPRESSION OF CONSTANS1* (*SOC1*), and *SHORT VEGETATIVE PHASE* (*SVP*) exhibited higher expressions in the bearing leaf (Figure 5); however, *EMBRYONIC FLOWER* (*EMF1*) was downregulated. Vernalization pathway genes *FRIGIDA*, *FCA*, and *SUPPRESSOR OF FRIGIDA4* (*SUF4*) were highly expressed in the bearing leaf. Photoperiod pathway genes *EARLY FLOWERING 4* (*ELF4*), *GIGANTEA* (*GI*), *LATE ELONGATED HYPOCOTYL* (*LHY*), and *FLOWERING LOCUS T* (*FT*) exhibited higher expressions in the bearing leaf. Interestingly, the phytochrome B (*PHYB)* gene was upregulated in non-bearing leaves. *BRASSINAZOLE-RESISTANT 1*, *GA20ox*, *GA INSENSITIVE DWARF 1B* (*GID1B*), *ETHYLENE INSENSITIVE 3* (*EIN3*), and the transcription factor PIF4 were upregulated in the bearing leaf. Genes such as *DELLA protein SLR1-like* and *GAIP-B-like*—a repressor of flowering and a GA signalling pathway—were higher expressed in the non-bearing leaf, most likely inhibiting the signal for flowering. Genes related to the circadian pathway, i.e., *EARLY FLOWERING 4* (*ELF4)*, *CIRCADIAN CLOCK ASSOCIATED 1 (CCA1)*, and *GI* were expressed at higher levels in the bearing leaf. Genes involved in the aging pathway such as *TOPLESS-related protein* and *SPL15* were also upregulated in the bearing leaf. Other genes involved in autonomous pathways such as *Flowering locus D* (*FLD*), *FPA*, *FCA*, and *FVE* presented higher expressions in the bearing leaf, indicating that they play significant roles in flowering in mango, most likely by inhibiting the floral repressor *FLC*. Moreover, upstream of *FLC* (*UFC*) a positive regulator of *FLC* was upregulated in the non-bearing leaf.

### 3.4 Functional annotation and GO term enrichment analysis

To obtain functional information on the DEGs, annotated biological and biochemical functional analyses were run using the WEGO database. Based on their GO classification, the differentially expressed transcripts were classified into three high-level categories: molecular functions, cellular components, and biological processes. To characterize gene function distribution at the macro level, we performed a GO enrichment analysis to determine the DEG functions. The results of the GO functional enrichment are shown in Figure 3. A total of 46,866 GO annotations were identified in different conditions. They were grouped into molecular, biological, and catalytic GO categories. For the biological process, the highest enrichment and number of DEGs were observed in the cellular protein metabolic process, followed by gene expression. For molecular function, purine nucleotide binding was a highly represented GO term, followed by binding and transporter activity. Active ion transmembrane transport was the least represented of the GO terms. These annotations provide an important resource for further investigation of the specific pathways involved in mango flower development.

### 3.5 Biochemical pathways analysis for bearing and non-bearing mango

**FIGURE 6:**
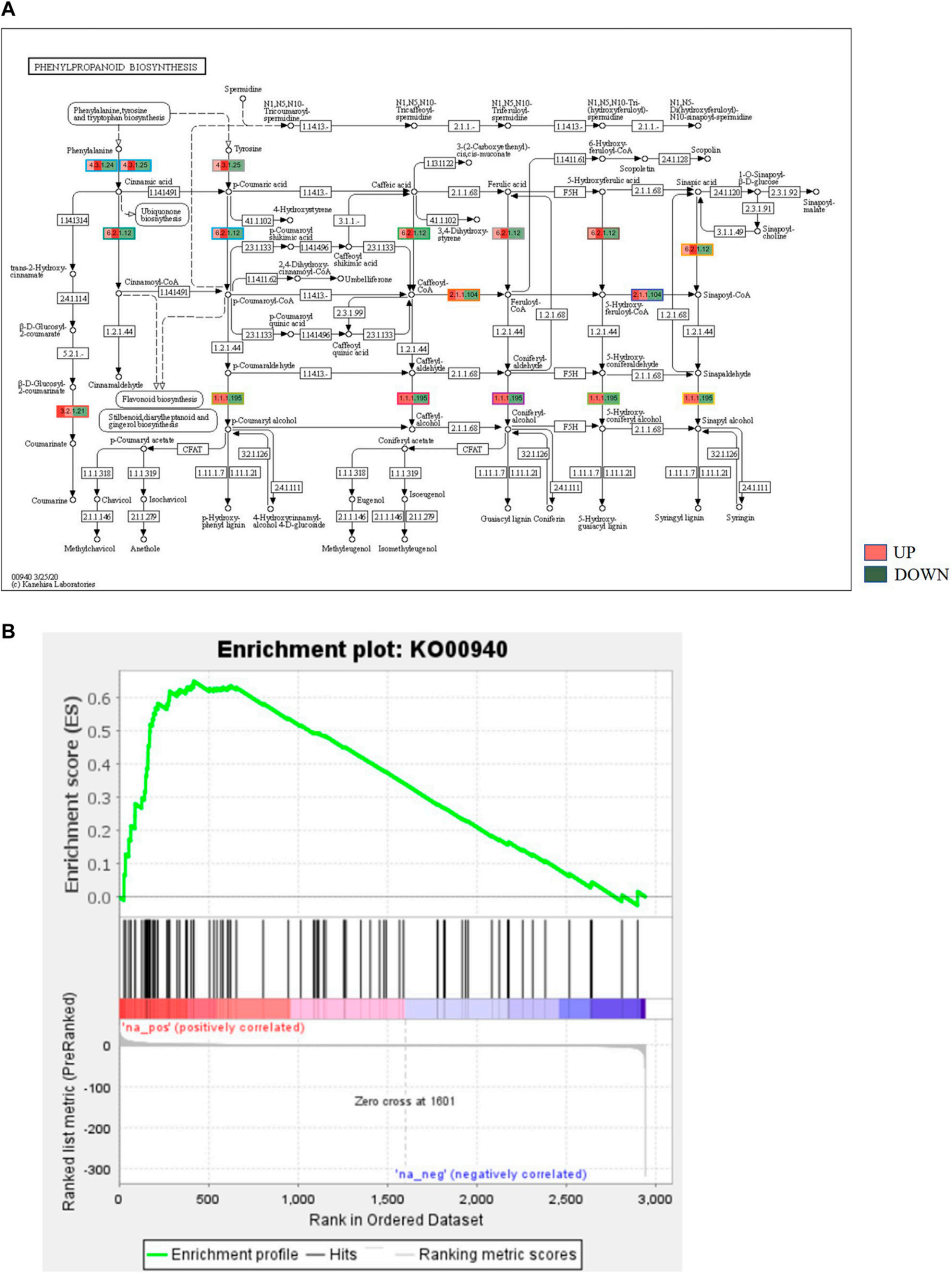
**(A)** KEGG binning analysis showing phenylpropanoid pathway up regulation in Dashehari non-bearing leaf. **(B)** Gene Set Enrichment Analysis (GSEA)—Upregulation of phenylpropanoid pathway genes in the non-bearing Dashehari compared to bearing leaf.

**FIGURE 7:**
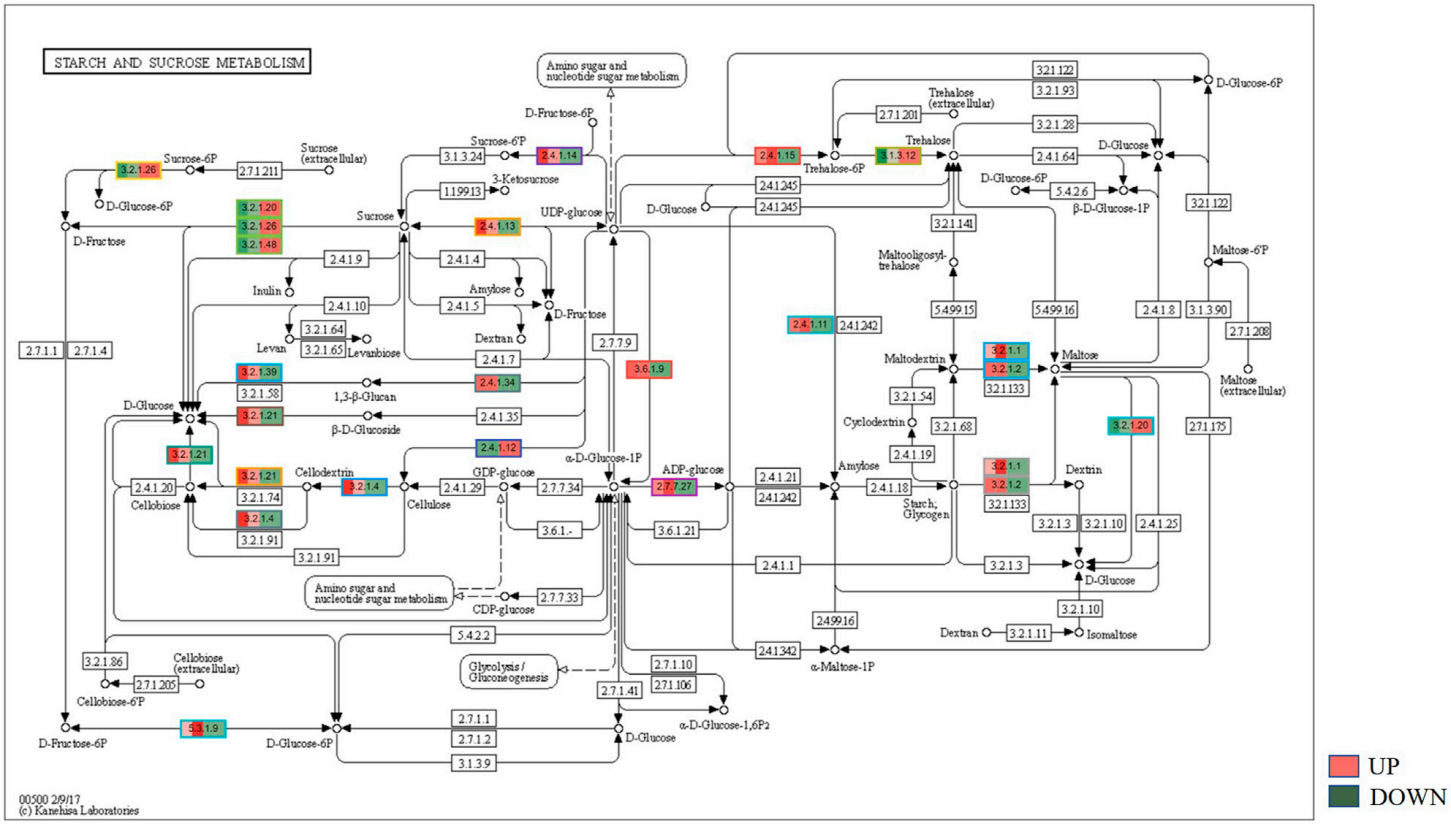
KEGG binning analysis shows-sucrose and starch pathway has shown up-regulation in Dashehari non-bearing leaf.

**FIGURE 8:**
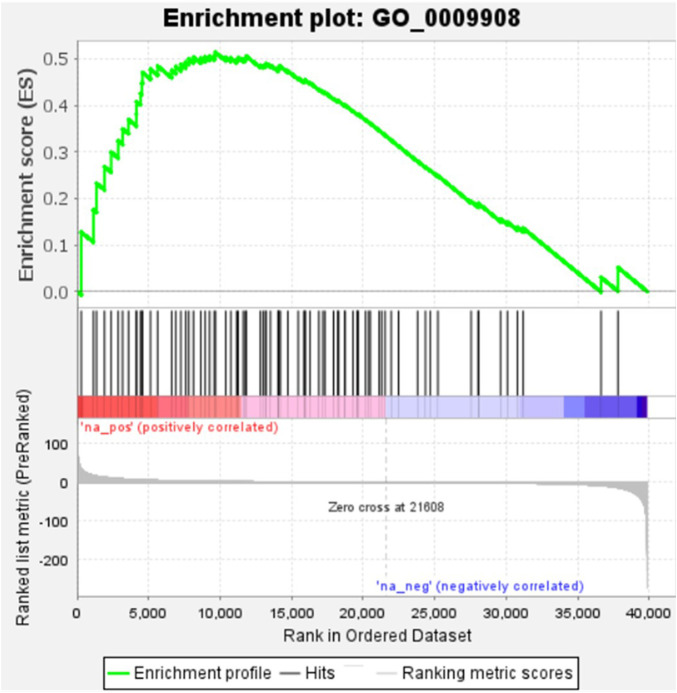
Gene Set Enrichment Analysis-Upregulation of flower development pathway genes in Dashehari inflorescence compared to bearing leaf.

KEGG, an alternative functional gene annotation system, assigns genes linked with biochemical pathways, based on Enzyme Commission (EC) numbers. Biochemical pathways were generated to further demonstrate the advantages of the obtained transcripts in identifying flower-related genes. This method predicted 139 pathways, 46 of which were enriched. 63 sequences were found to be related to the flavonoid biosynthesis pathway. Phenylpropanoid biosynthesis (Figure 6A) and starch and sucrose metabolism pathways (Figure 7) were linked with 158 and 606 transcripts, respectively. These pathways were activated in non-bearing leaves. Moreover, in GSEA analysis, a higher enrichment score for phenylpropanoid biosynthesis was observed in non-bearing leaves (Figure 6B). Additionally, flower development pathways showed higher enrichment scores in the inflorescence as compared to the leaf (Figure 8). Furthermore, 208, 129, and 148 transcripts linked to glyoxylate and dicarboxylate metabolism, tryptophan metabolism, and beta-alanine metabolism pathways, respectively, were downregulated in the non-bearing leaf. The induction of flowering is facilitated by higher nitrogen levels. Eighty transcripts were found to be associated with nitrogen metabolism, with higher expressions in the bearing leaf, of which glutamine synthetase cytosolic isozyme 1 and glutamate dehydrogenase 1 exhibited the highest transcript expressions.

### 3.6 Validation of genes involved in flowering through qRT-PCR

**FIGURE 9:**
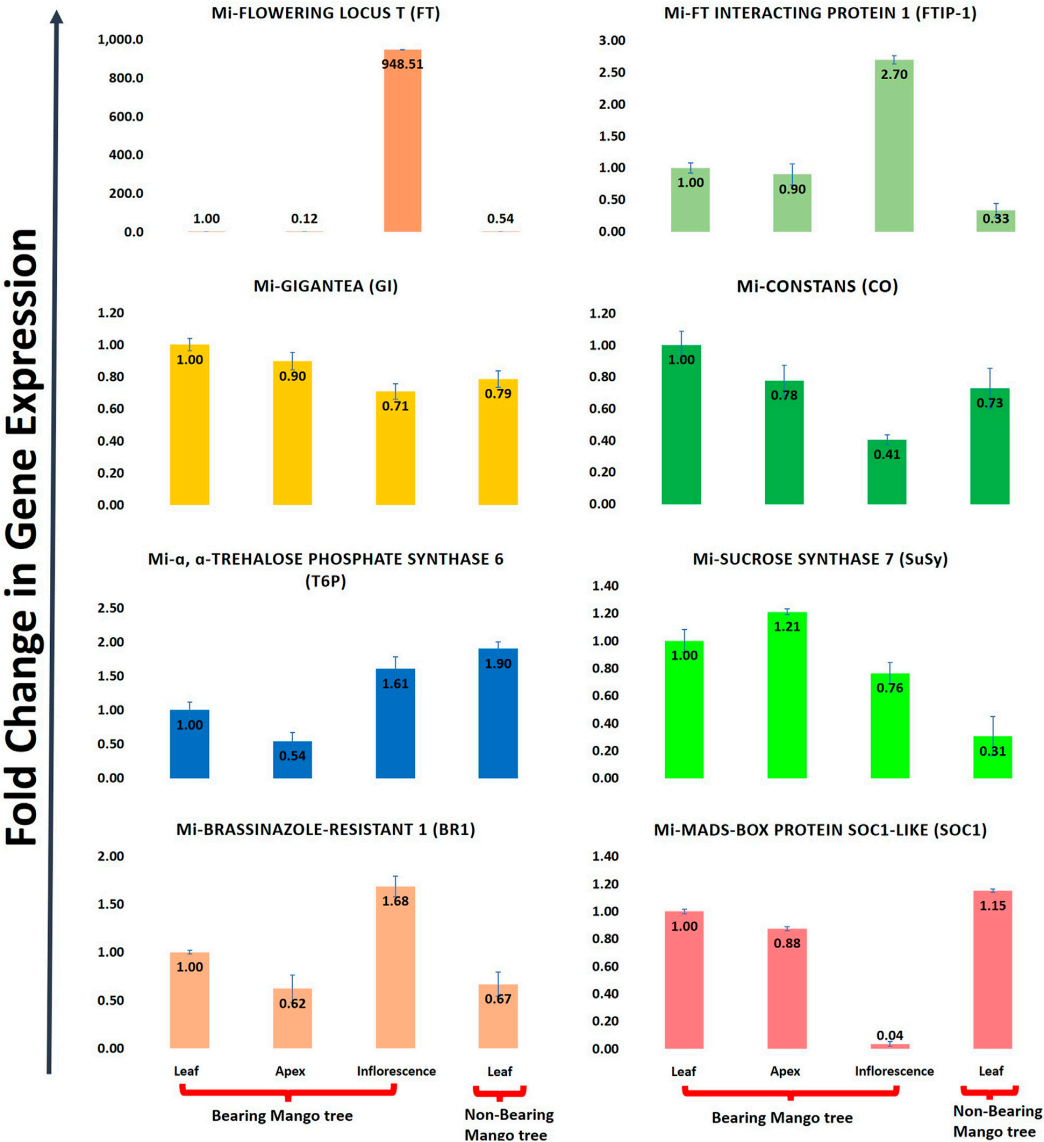
Quantitative real time (qRT-PCR) expression of eight flowering genes (FT, FTIP, GI, CO, T6P, Sucrose synthase, BR1, SOC1) in leaf, apex, inflorescence and non-bearing leaf of Dashehari. Mi-Actin is used as an internal reference. The fold change data is normalized to Dashehari bearing leaf (DL). Tissue types compared are Dashehari bearing leaf vs. Dashehari apex, Dashehari bearing leaf vs. Dashehari inflorescence, Dashehari bearing leaf vs. Dashehari non-bearing leaf, Dashehari apex vs. Dashehari inflorescence. Error bars represents ± Standard error with n = 3.

*Mangifera indica* cv. Dashehari transcripts corresponding to FT, FTIP, GI, CO, T6P, SUCROSE SYNTHASE, BR1, and MADS-SOC1 were queried with qRT-PCR assays to validate the *in silico* RNA-seq results. Interestingly, most of the results corroborated the differential count values of the high-throughput data. For instance, a ∼948-fold increase in FT gene expression was observed in the inflorescence compared with leaf tissue, which was concomitant with the increase in FTIP-1, validating the assistance of FTIP to FT transport from the leaves to inflorescence (Figure 9). The expressions of FT and FTIP were downregulated by one-third in non-bearing leaf tissue. Sucrose synthase 7 was more highly expressed in the bearing leaf compared with the non-bearing leaf, further supporting sucrose synthase control of sugar signalling to induce flowering; it may function as a signalling factor in the development of the Shoot Apical Meristem (SAM) into flowers (Cho et al., 2018). The sucrose synthase signal for flowering may be mediated by T6P, which was upregulated in the inflorescence (Figure 9). To control flowering, BRASSINAZOLE-RESISTANT1 (BR1) binds to cis-elements in the FLOWERING LOCUS D (FLD) promoter. Accordingly, BR1 was upregulated in the leaves and inflorescences of bearing trees, and downregulated in non-bearing tree leaves. The T6P level is an indicator of high sucrose levels, and the expression of TPS1 in the SAM of Arabidopsis plants induces early flowering, further supporting the proposed roles of sucrose and T6P in SAM flowering (Wahl et al., 2013).

## 4 Discussion

Flowering in mango is a complex phenomenon, presenting difficulties for physiologists, breeders, and growers to assess the expected yield every year. Several concepts have been proposed by various researchers; however, none of them hold for at least one variety because mango flowering is influenced by a variety of factors (Sandip et al., 2015). Flowering is an aspect of mango reproductive biology that has captivated the interest of researchers all over the world. To comprehend the molecular basis for traits that have not been well defined, *de novo* transcriptome assembly is vital. It has already been employed in studies on differential expression of a variety of perennial fruit crops, including litchi (Li et al., 2015), citrus (Shalom et al., 2014), olive (Turktas et al., 2013), apple (Guitton et al., 2012; Guitton et al., 2016) and the number of studies has increased enormously owing to improvements in NGS toolkits. Here, Illumina-based RNA sequencing was applied to profile the mango’s transcriptome, with a focus on detailed analysis to identify genes and pathways that control flowering time. RNA-seq data from five tissue samples of Dashehari (bearing leaves, non-bearing leaves, apex, inflorescence, and malformed inflorescence) were assembled into 67,915 transcripts, with 25,776 unigenes. Among these, 43,373 transcripts were assigned a specific or general function based on a comparison against sequences in the “nr”, KEGG, and GO databases. Subsequently, DEGs analysis was performed by comparing different tissues of Dashehari from the bearing and non-bearing leaf, apex, and inflorescence. These had 30,430 transcripts in common, indicating that the majority of unigenes were constantly expressed across all tissues. During the flower development process, the majority of transcripts were enriched in the synthesis of fatty acids, microtubule-based processes, floral morphogenesis, regulation of flower development, and flower development. Similarly, Wang et al. (2018) discovered that most DEGs involved in biological processes at various developmental stages (from bud stage to early flowering) of *R. pulchrum* were associated with the fatty acid biosynthetic process, protein polymerization, and the microtubule-based process (Wang et al., 2018). We identified 428 transcription factors (TFs) belonging to different plant TF families in mango. The most abundant TFs included MYB, bHLH, WRKY, NAC, and ERF. The mango transcriptome was mapped to 139 pathways using KEGG pathway analysis. An integrated network that involves photoperiod, ageing, circadian clock, and vernalization pathways regulates mango flowering, most likely by its integration on trehalose-6-phosphate (Figure 5). T6P metabolism has been shown to influence inflorescence architecture in maize, Arabidopsis, and many other plant species (van Dijken et al., 2004; Satoh-Nagasawa et al., 2006).

### 4.1 Gigantea, constans, suppressor of constans, flowering locus T, early flowering 4, and FT-interacting protein—controlling photoperiod and circadian clock interactions in the bearing mango tree leaf for increased expression and movement of FT in the inflorescence

GIGANTEA promotes flowering, along with CONSTANS (CO) and FLOWERING LOCUS T (FT). Flowering thereby results from the accumulation of CO protein. In Dashehari, GI is highly expressed in the bearing leaf and inflorescence when compared with the non-bearing leaf. Petunia × hybrida plants, which lack GI1 function, exhibited fewer floral buds and smaller flowers than the wild-type, when grown in long-day conditions (Brandoli et al., 2020). CONSTANS (CO) coordinates the circadian clock and phytochrome inputs into leaves, acting as a major regulator that triggers the generation of the mobile florigen hormone FT, which stimulates flower differentiation. In Dashehari bearing leaves, FT was found to be upregulated. According to Suárez-López et al. (2001), CO encouraged the expression of FT in Arabidopsis thaliana when long days were induced. The integration of photoperiod perception, circadian regulation, and flowering was facilitated by the circadian clock gene EARLY FLOWERING 4 (ELF4), and the expression of ELF4 were upregulated in bearing mango leaf tissue. In order to facilitate floral transition, a MADS box transcription factor and SOC1 participate as integrating nodes in the photoperiod, temperature, aging, and gibberellin pathways. SOC1 was found to be upregulated in the apex and bearing leaf of Dashehari mango. Similar observations by Liu et al. (2020) indicate that PbSOC1d and PbSOC1g performed functions in pear flowering development. In order to control flowering, the photoperiod regulatory centre gene CO interacts with the promoter of the FT gene, leading to florigen signal activation. Secondly, SOC1 and Leafy (LFY) help the FT gene to integrate flower-induced signals from the photoperiod, autonomous, and vernalization pathways (Yoo et al., 2005). Additionally, it has been proposed that the phloem system plays a role in the long-distance transport of FT protein from the leaves to the shoot apex (Tsuji, 2017). To begin flower development in the SAM, FT interacts with the bZIP transcription factor FLOWERING LOCUS D (FD), which in turn activates downstream floral meristem identity genes such as APETALA1 (AP1). In this study, one homolog of FT exhibited expression in the bearing leaf and no expression in the non-bearing leaf, demonstrating that the FT gene is necessary for flowering. Additionally, the FT gene showed higher levels of expression in the apex and inflorescence, relative to the leaf, indicating the movement of FT transcripts from leaves to buds in accordance with the theory that florigen is produced in the leaves and then moves to the apex and inflorescence to assist floral transition (Corbesier et al., 2003; Putterill and Varkonyi-Gasic, 2016). For FT protein transport to cause flowering in Arabidopsis, FTIP1 is a crucial regulator. Due to delayed FT transport to the shoot apex, loss of function of FTIP1 results in late flowering under long days (Liu et al., 2012). In our study, FTIP-1 was upregulated in the inflorescence compared with DL; however, DLNB exhibited an FTIP-1 expression that was higher than that of DL.

### 4.2 Vernalization pathway interacts in a positive manner to upregulate flowering in bearing mango leaves

By promoting the expression of the floral repressor FLC, FRI suppresses flowering. Vernalization counteracts the effect of FRI by accelerating flowering through suppression of the FLC expression. By interacting with FRIGIDA LIKE 1 (FRL1) at the N-terminus, and SUPPRESSOR OF FRIGIDA 4 (SUF4), FRIGIDA ESSENTIAL 1 (FES1), and FLC EXPRESSOR (FLX) at the C-terminus, the FRI protein functions as a scaffold to generate a transcription activation complex (Choi et al., 2011). Additionally, this FRI complex interacts with HAM1, SWR1-C, and UBC1 to form an FRI super complex, which in turn modifies the chromatin structure and promotes FLC mRNA transcriptional activation. Additionally, the FRI protein degrades when exposed to cold, indicating that FRI also contributes to the downregulation of FLC during vernalization (Hu et al., 2014). In contrast, FRI, SUF4, and FLC EXPRESSOR were found to be upregulated in bearing mango leaves here. However, MADS-box SVP, a transcriptional repressor that inhibits floral transition in the autonomous flowering pathway, was highly expressed in the non-bearing leaf. *VERNALIZATION INSENSITIVE 3 (VIN3)* plays a central role in vernalization by mediating the initial transcriptional repression of the homeotic floral repressor FLC. VIN3, was upregulated in the bearing mango leaf and inflorescence. Additionally, several genes influencing flowering in a positive manner, including FCA, FPA, FLD, AGAMOUS-MADS2, and AGAMOUS-FUL-L, were highly expressed in the bearing leaves. SPL15 is expressed in the vegetative meristem before floral induction, throughout meristem development, and in the inflorescence (Hyun et al., 2017). In this study, SPL15 was expressed in the bearing leaf, apex, and inflorescence of mango. Embryonic flower 1 delays the transition from the vegetative to the reproductive phase of flower development as well as the onset of flowering. EMF1 was upregulated in the non-bearing leaves, indicating the inhibition of florigen production in the leaf tissue. In Arabidopsis, the FLC gene is located in the middle of the hierarchy: between UPSTREAM OF FLOWERING LOCUS C (UFC) and DOWNSTREAM OF FLOWERING LOCUS C (DFC) (Finnegan et al., 2004; Aljaser et al., 2021). Vernalization inhibits the expression of the UFC gene, without the involvement of FLC (Finnegan et al., 2004). As a result, the UFC gene’s proximity to FLC in Arabidopsis serves as an early signal of FLC homologue presence. By virtue of the fact that UFC was higher in the non-bearing leaves, this is a proxy for non-identification of FLC in our dataset, suggesting FT gene suppression in the non-bearing mango leaves.

### 4.3 Hormonal pathway interplay in flowering induction

Hormones are crucial for the growth and development of a plant. The control of biennial bearing and flowering appear to be mediated by autonomous GA pathways in mango (Nakagawa et al., 2012). The current study identified six transcripts of the gibberellin receptor GID1B-like and DELLA protein GAIP-B-like in the GA pathway in the bearing leaves; their expression levels varied during flowering, suggesting that these genes may be crucial for controlling flower formation. The hormones ethylene and abscisic acid, which suppress FT, cause plants to delay flowering. Non-bearing leaves were observed to exhibit higher levels of SUPPRESSOR OF ABI3 while EIN3 was higher in the bearing leaf. For floral initiation, assimilates must be diverted from shoot apices to the floral primordial (Kalayanaruk et al., 1982). Kalayanaruk et al. (1982) and Chacko (1984) found a direct correlation between mango flowering and carbohydrate reserves; T6P may play a crucial role (Turktas et al., 2013). Furthermore, sucrose synthase 4 expression increased in the mango shoot apex in our study, suggesting that it may play a crucial role in the development of the SAM (Stein and Granot, 2019) into the inflorescence. The DGE enrichment study also revealed genes related to the auxin signalling pathway, cytokinin biosynthesis process, and nitrogen metabolism. Cytokinins have been shown to play crucial functions in the floral development process in Arabidopsis (Corbesier et al., 2003). During floral induction, a higher level of cytokinins is necessary in mango stem buds (Naphrom et al., 2004); it was discovered that cytokinin expression was upregulated in the bearing mango tree leaves. BRASSINAZOLE-RESISTANT1 (BZR1), whose conformation is changed by the cyclophilin (CYP20-2), binds cis-elements in the FLD promoter to control flowering (Zhang et al., 2013). In our findings, it was discovered that BZR1 was upregulated in the bearing leaf.

## 5 Conclusion

The Dashehari transcriptome and comparison of bearing *vs.* non-bearing mango tree tissues performed in this study underpin the role of signalling hormones integration with florigen in the leaf tissue and further mobilization from the leaf tissue to the apex and inflorescence. The high expression of candidate genes such as T6P, a well-known sugar signal, may well be a subject for critical analysis, where one can spray different carbohydrate formulations to induce flowering by altering the plant physiology, rather than spraying a prohibited gibberellin inhibitor paclobutrazol.

## Data Availability

The data presented in the study are deposited in the DDBJ/EMBL/GenBank repository, accession number for de novo assembly is GKCW00000000 under BioProject accession number PRJNA879302.
