# Supplementary figures and images for: Comparative transcriptomics in alternate bearing cultivar Dashehari reveals the genetic model of flowering in mango
Source: Front Genet. 2023 Jan 10;13:1061168. doi: 10.3389/fgene.2022.1061168 (PMC9871253; doi:10.3389/fgene.2022.1061168)

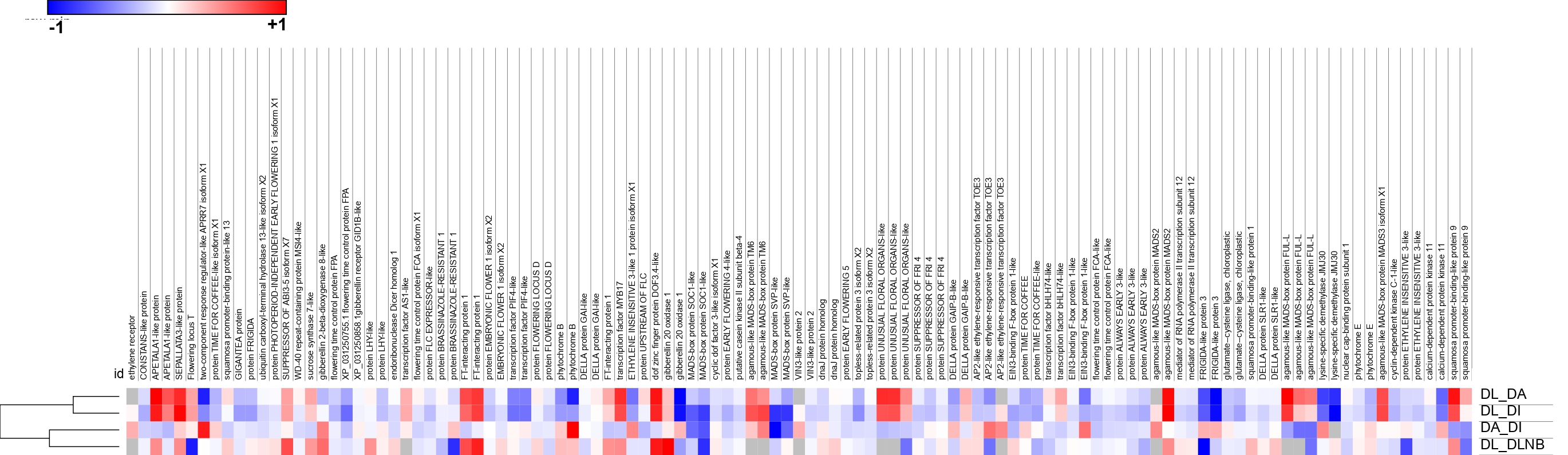

Supplement: Supplementary file 1 [file Image3.JPEG]

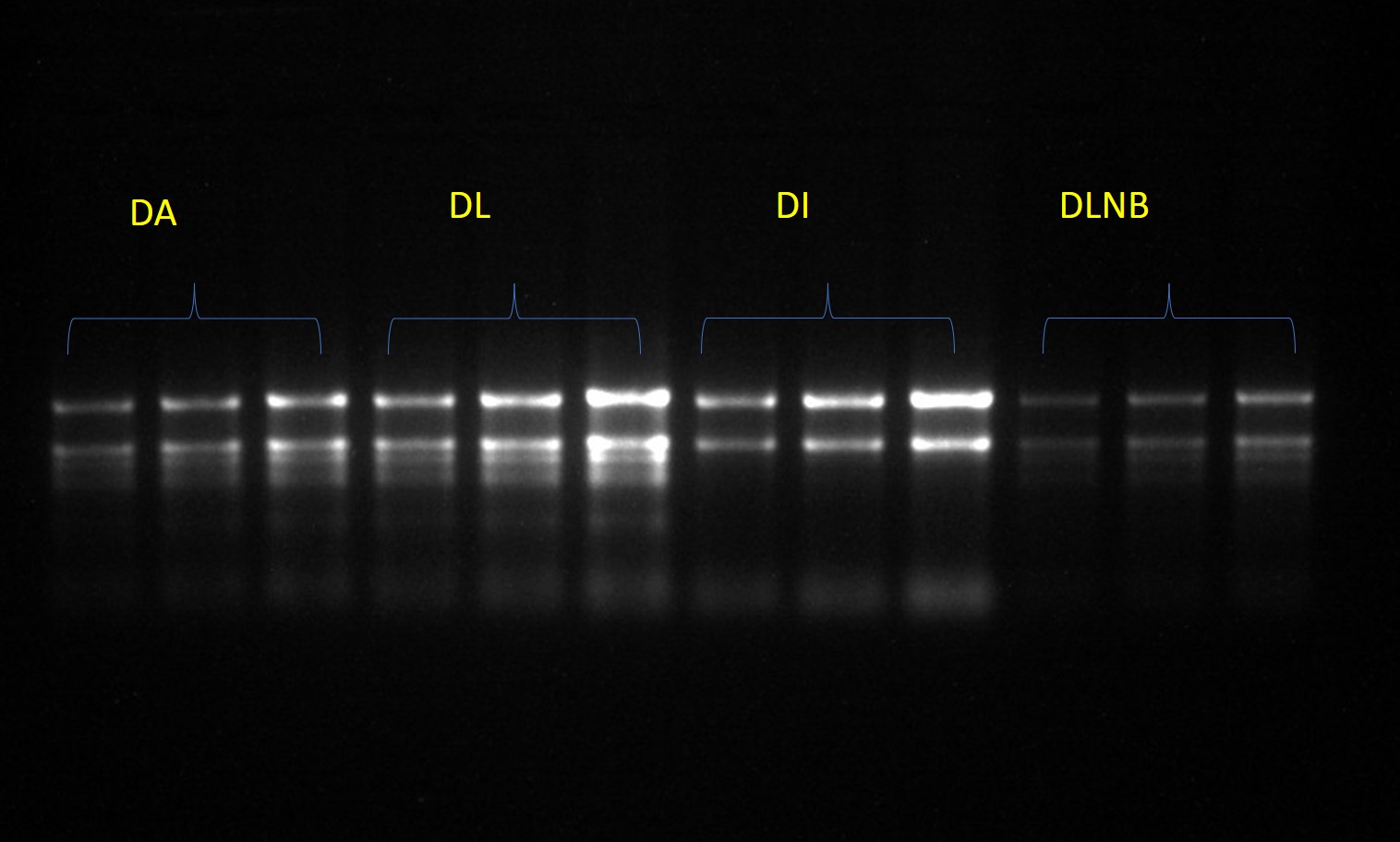

Supplement: Supplementary file 3 [file Image1.JPEG]

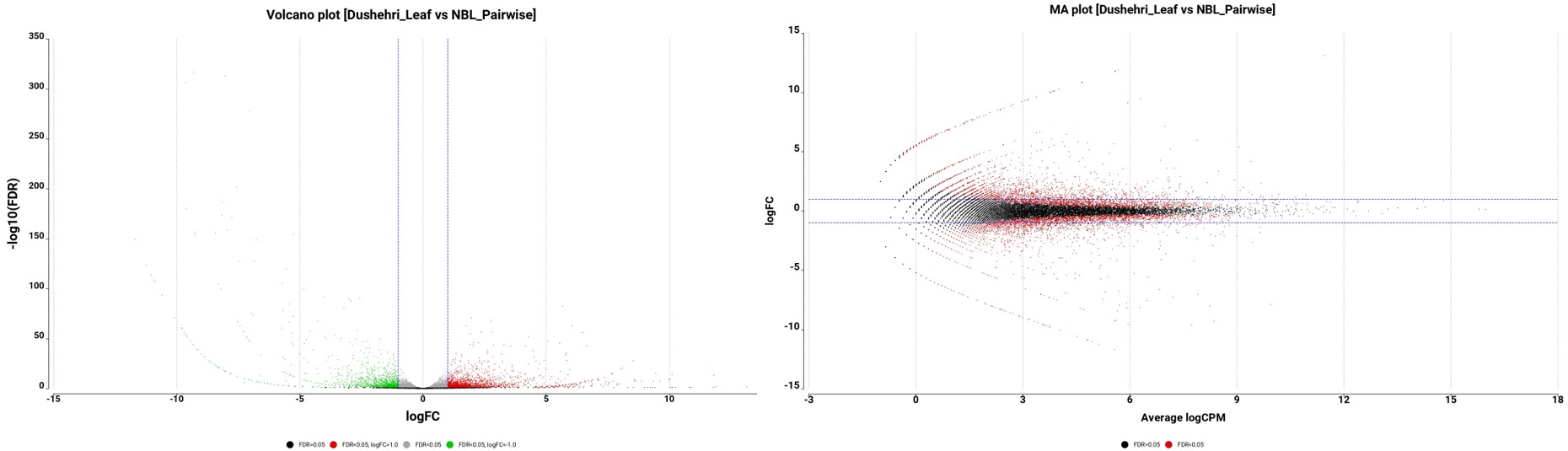

Supplement: Supplementary file 4 [file Image2.JPEG]
