# Supplementary material for: Comparative transcriptomics in alternate bearing cultivar Dashehari reveals the genetic model of flowering in mango
Source: Front Genet. 2023 Jan 10;13:1061168. doi: 10.3389/fgene.2022.1061168 (PMC9871253; doi:10.3389/fgene.2022.1061168)
Supplement: Supplementary file 2 [file Table1.docx]

**Supplementary Table 1**. Summary of mango tissue types, RNA-seq clean and raw reads

| **Mango cv. Dashehari tissues** | **Library** | **Raw Reads pair** | **GC %** | **Surviving Reads** |
| --- | --- | --- | --- | --- |
| Leaf (DL-80) | LIB29211 | 34,540,354 | 43 | 25,740,968 (74.52%) |
| Apex (DA-12) | LIB29212 | 28,808,884 | 47 | 22,537,536 (78.23%) |
| Inflorescence (DI-29; DI-73) | LIB29213; LIB29214 | 25,007,480; 28,980,802 | 47; 45 | 19,346,111 (77.36%); 22,631,709 (78.09%) |
| Non-bearing Leaf (DLNB-18; DLNB-19) | LIB29217; LIB29218 | 29,875,796; 27,195,035 | 43; 43 | 22,837,761 (76.44%); 21,171,900 (77.85%) |

**Supplementary Table 2:** Oligomer sequences

| 1 | CONSTANS | Left primer | gaagcagtggtcgttcaccaga |
| --- | --- | --- | --- |
|  |  | Right primer | ggttgaagatggccctgaaatg |
| 2 | GIGANTEA | Left primer | gggattgcatctgtgctttgtg |
|  |  | Right primer | cggctgaagaactcaacggaat |
| 3 | SUCROSE SYNTHASE | Left primer | gcagtaaggaaaggcctggaca |
|  |  | Right primer | aattcctcaatggcagggtgaa |
| 4 | FTIP-1 | Left primer | cctggaagcagacaggaggaaa |
|  |  | Right primer | tgtttccagagctgctttgctg |
| 5 | FT | Left primer | agctatggccacaagctggaag |
|  |  | Right primer | tttaaggtgggggtcacttgga |
| 6 | T6P | Left primer | gaggatgccgatccagattttg |
|  |  | Right primer | cctcgacaagattctgcccact |
| 7 | BRI | Left primer | ccactgttgattccggtcactg |
|  |  | Right primer | actctggatttcgccccttctc |
| 8 | MADS-SOC1 | Left primer | ggatgcaaacgacaacagatcg |
|  |  | Right primer | tcgaacccaaatcttgccctaa |
| 9 | ACTIN | Left primer | gatgcccagaagtcctcttcca |
|  |  | Right primer | caagggctgtgatttccttgct |

**Supplementary Table 3**. The number of differentially expressed genes in different comparisons

| **Bearing Leaf vs other tissues** | **Total differentially expressed transcripts** | **Upregulated (≥+4fold change)** | **Down-regulated (≤-4-fold change)** |
| --- | --- | --- | --- |
| DL_DLNB | 37236 | 1603 | 955 |
| DL_DA | 38579 | 7923 | 8790 |
| DA_DI | 38873 | 1794 | 263 |
| DL_DI | 39852 | 7773 | 6143 |
